# A comparative analysis of toluidine blue with frozen section in oral squamous cell carcinoma

**DOI:** 10.1186/1477-7819-10-57

**Published:** 2012-04-16

**Authors:** Montasir Junaid, Moaz M Choudhary, Zain A Sobani, Ghulam Murtaza, Sadaf Qadeer, Naeem S Ali, Mumtaz J Khan, Anwar Suhail

**Affiliations:** 1 Department of Otolaryngology, Head and Neck Surgery, Jinnah Medical And Dental College Hospital, SR-6, Sector 7/A, Korangi Industrial Area, Karachi - 74800 Karachi, Pakistan; 2 Student, Medical College, Aga Khan University, Karachi, Pakistan; 3 Department of Surgery, Aga Khan University, Karachi, Pakistan; 4 Section of Otolaryngology/Head and Neck Surgery, Department of Surgery, Aga Khan University, Karachi, Pakistan; 5 Section of Otolaryngology/Head and Neck Surgery, Department of Surgery, Aga Khan University Hospital, Ocean Road, Dar es Salaam 2289, Tanzania; 6 Head and Neck Institute, Cleveland Clinic Foundation, Cleveland, Ohio, USA

## Abstract

**Background:**

Surgical excision of the primary tumor with safe margins remains the mainstay of treatment for oral cavity squamous cell carcinoma (OSCC). The standard of care for assessment of intraoperative margins is frozen section histopathology. Unfortunately the facility is not available at most centers in limited resource countries. Toluidine blue, a metachromatic dye, has been well described in clinical identification of malignant and premalignant lesion in the oral cavity. Considering this we decided to explore intraoperative use of toluidine blue staining, in comparison with frozen sections, for the assessment of tumor-free margins.

**Methods:**

After obtaining clearance from the in-house ethical review committee, a prospective study was conducted at Aga Khan University Hospital, Karachi, from August 15, 2009 to March 14, 2010. A sample of 56 consenting patients with biopsy-proven OSCC were included in the study, giving us 280 tumor margins. Margins were analyzed using toluidine blue staining and frozen section histopathology. A receiver operator curve (ROC) was then applied to compare assessment of margin status by toluidine blue and frozen section.

**Results:**

Of the 280 examined margins 11 stained positive with toluidine blue, three were positive on frozen section biopsy, and three were positive on final histopathology. Toluidine blue staining had sensitivity and specificity of 100% and 97%, respectively. The diagnostic accuracy of toluidine blue was found to be 97.1% with a positive predictive value (PPV) of 27.2% and a negative predictive value (NPV) of 100%.

**Conclusions:**

Toluidine blue can be used as an effective screening modality for the assessment of intraoperative margins in resource limited environments and reducing the number of frozen section biopsies performed. Further by providing real-time clinical information within minutes it can reduce indirect costs such as operating room time. It may also be used as an ad hoc for frozen section biopsies where frozen section facilities are available.

## Background

When assessing the incidence and prevalence of cancers the International Agency for Research on Cancer, an arm of the World Health Organization (WHO), ranked head and neck cancers as the sixth most common malignancies in males [1]. Contributing to the same database, Bhurgri *et al.*; while reviewing their data from Karachi, Pakistan found a higher burden of disease [2]. In the local data, head and neck neoplasms accounted for the highest number of malignant neoplasm in males (age standardized rate (ASR) of 22.5 per 100,000) and the second highest number in females (ASR of 20.4 per 100,000) [2]. Given the prevalent cultural practices of chewing betel nuts and tobacco, it came as no surprise that the most frequent site for these neoplasms was the oral cavity and squamous cell carcinoma the most predominant subtype [2,3]. The alarmingly high incidence of oral cavity and squamous cell carcinoma (OCSCC) is indeed a major health issue for a third world country, with a staggering economy and a fee for service health structure. The situation is further marred by the absence of screening protocols.

The mainstay of treatment of OSCC involves surgical excision of the primary tumor with safe margins [4]. This may be augmented by radiation therapy with or without chemotherapy. However intraoperative assessment of tumor margins remains a major health issue. Methods of identifying tumor-involved margins include visualization and simultaneous palpation of the resected margins but with a chance that microscopic disease will be left behind. Frozen section biopsies are used as the standard of care in this regard in developed countries. Given the status of healthcare systems in limited resource settings like ours, it is not available in a majority of centers. On an average, the cost of five frozen margins in a patient is approximately US$75. Adding to this is the fact that over half the population lives on less than US$2 a day, making the service where available out of reach for a majority of patients.

Toluidine blue is an easily available, economical, metachromatic dye known to bind DNA of dividing cells. It has previously been described to stain malignant and premalignant cells but not normal mucosa [5-7]. Multiple studies have reported the use of toluidine blue as a screening tool for oral malignant and pre-malignant lesions [8-12], with sensitivity and specificity between the ranges of 93.5% and 97.8% and 73.3% and 92.9%, respectively [13]. Given this background we decided to test toluidine blue for assessment of intraoperative tumor margins after the excision of primary oral cavity tumor.

## Method

After ethical clearance from the institutional ethics review committee, a prospective study at The Aga Khan University Hospital, Karachi, Pakistan was conducted from August 15, 2009 to March 14, 2010 on 56 consenting patients with biopsy-proven OCSCC, undergoing primary excision. Patients with prior history of head and neck malignancy, tumors of the oro-pharynx or having previously undergone treatment (surgery and/or radio-/chemotherapy) for the current oral squamous cell carcinoma were excluded from the study as increased inflammation and scar tissue could possibly lead to errors while interpreting the staining results. The patients were recruited regardless of the age, gender, ethnicity, and tumor stage.

All procedures were performed by experienced head and neck surgeons with assistance from senior residents. The operative protocol was essentially the same with minor differences due to the individual tumor status and attending surgeon's preference. After excision of the primary tumor a trained senior resident stained the tumor margins as per the defined protocol. Once the senior was done painting the margins and had recorded his findings, the primary surgeon took frozen biopsies from clinically suspicious areas regardless of staining pattern as per institutional protocol. In addition if any margin stained positive with toluidine blue the primary surgeon was requested to take a frozen section biopsy from the area. Each frozen section biopsy was sent in a different container with labeling of the site from which it was obtained. A log was maintained of the staining results. The senior resident and the histopathologist were blinded to each other's findings (histopathologist for the staining pattern, senior resident for frozen results). However the staining may have persisted after processing and may have had an influence on the histopathologist's decision. The results of the frozen section biopsy were directly conveyed to the attending physician via the operating room (OR) phone. The senior resident performing the staining was not present in the OR at the time these results were conveyed. The results where then compared and contrasted with the findings of frozen section biopsy and final histopathology by another author. No intraoperative action, other than frozen section biopsy, was taken on the basis of positive staining margins on toluidine blue, due to the inherent surgical protocol of our institution. However if a margin was declared positive on frozen section histopathology it was further excised and sent for frozen section again till the time it came out negative.

### Staining procedure

One per cent toluidine blue solution, as described by Meshberg *et al. *was kept in the OR. Once the primary tumor was excised a designated senior resident irrigated the tumor margins with normal saline, followed by 1% acetic acid. The margins were then gently dried with gauze and painted with the 1% toluidine blue solution with a cotton swab. The stain was left in place for 30 s after which the tissue was once again irrigated with normal saline followed by 1% acetic acid. The staining patterns were observed and noted by the senior resident independently. Margins which stained dark blue were considered to be positive, whereas those which stained light blue or did not take the stain at all were considered negative. In order to establish staining patterns we used simultaneous frozen sections in two pilot patients and revealed the results to the interpreter during the surgery, the results of which were not included in the study.

### Statistical analysis

The data were entered and analyzed using Statistical Package for Social Sciences (SPSS) version 17 (IBM Corporation). Means with standard deviations were calculated for all continuous variables while proportions and percentages were calculated for categorical variables. A 2 × 2 table was constructed from the acquired data, and the measures of yield (sensitivity and specificity) and reliability (negative predictive value and positive predictive value) were calculated using Microsoft Excel 2010 (Microsoft Corporation). Separate 2 × 2 tables were constructed using SPSS for each category of T stage and tumor site to calculate above measures. A receiver operator curve (ROC) was then applied to compare the margin status between toluidine blue and frozen section.

## Results

Out of 56 patients the majority (75%) were men. About 280 margins were sampled from this pool of patients. Mean age of the patients was 50.07 ± 15.73 years. Buccal mucosa tumors accounted for 41% (*n *= 23) of the lesions. This was followed by the tongue 23.2% (*n *= 13), lower alveolus 12.5% (*n *= 7), retro-molar trigone 8.95% (*n *= 5) and the floor of the mouth 5.3% (*n *= 3). When considering the extent of the lesion 19.6% (*n *= 11) patients were staged as T1, while 33.9% (*n *= 19) were staged as T2, 19.6% (*n *= 11) as T3 and 26.7% (*n *= 15) as T4.

Of the 280 examined margins 11 stained positive with toluidine blue. However of these 11 margins three were true-positives, that is positive on frozen section biopsy and/or final histopathology (Table 1). There were no false-negatives, that is toluidine blue was able to identify all true positive margins. While all the positive cases were picked up on staining with toluidine blue indicating a sensitivity of 100%, 8/280 (2.8%) false-positives were also identified indicating a specificity of 97%. The diagnostic accuracy of toluidine was found to be 97.1% with a positive predictive value (PPV) of 27.2% and a negative predictive value (NPV) of 100%.

**Table 1:** A 2 × 2 table showing the comparison of results of toluidine blue with frozen section

		Frozen section/Final histopathology		Total
	Margin Status	+ve	-ve	
Toluidine Blue	+ve	3	8	11
	-ve	0	269	269
Total	3	277	280	

When applying the ROC analysis we found that the low positive predictive value was significant (*P *= 0.004).

On stratified analysis, the sensitivity and NPV remained unchanged across all the strata of T stage and tumor sites. In contrast, specificity and PPV were found to decrease with larger stage III and IV tumors; however they did not fall below acceptable levels (Table 2). In our study the results of the frozen section biopsies were in concordance with the findings on final histo-pathological examination. (Table 3) Hence comparison of toluidine blue staining to frozen section biopsies and histopathology revealed the same results. We did not come across any dysplasia on final histopathology.

**Table 2:** Showing the yield (sensitivity and specificity) and reliability (PPV and NPV) of toluidine blue as compared to frozen section biopsy and histopathological examination

		Sensitivity	Specificity	PPV	NPV
**Overall**		100%	97.1%	27.2%	100%

**T-Stage**					
T-I		-	98.1%	-	100%
T-II		100%	100%	100%	100%
T-III		100%	98.1%	50%	100%
T-IV		100%	91.8%	14%	100%

**Tumor**	**Site**				
Buccal	mucosa	100%	97.3%	25%	100%
Tongue		100%	98.4%	50%	100%
RetromolarTrigone		-	96%	-	100%
Lower	alveolus	100%	94.1%	33.3%	100%
Upper	alveolus	-	96%	-	100%
Floor of mouth		-	100%	-	100%

*PPV *positive predictive value, *NPV *negative predictive value

**Table 3:** Illustrating the 11 cases of positive staining margins with toluidine blue compared to the frozen section biopsy and final histopathology results

**No**.	Site of primary tumor	T staging	Frozen section biopsy results	Final histopathology results
1	Tongue	III	+ve	+ve
2	Retromolar trigone	III	-ve	-ve
3	Buccal mucosa	IV	+ve	+ve
4	Buccal mucosa	IV	-ve	-ve
5	Buccal mucosa	IV	-ve	-ve
6	Buccal mucosa	IV	-ve	-ve
7	Tongue	IV	-ve	-ve
8	Lower alveolus	IV	+ve	+ve
9	Lower alveolus	IV	-ve	-ve
10	Lower alveolus	IV	-ve	-ve
11	Upper alveolus	IV	-ve	-ve

**Figure 1:**
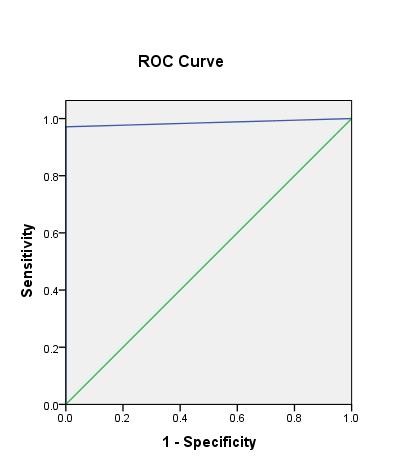
**Receiver operator curve showing the area under the curve for toludine blue at different levels of specificity 98.6% (AUC: 0.986, 95% CI: 0.968-1, p-value: 0.004**. The green and blue lines represent thediagonal and toluidine blue respectively.

As per ROC analysis, for a randomly selected margin positive case, there is a 98% chance that toluidine-predicted probability of margin positivity will be higher than a randomly selected margin negative case. This can also be explained that the probability of a toluidine positive case to become positive on histopathology by chance alone is 0.4% (AUC: 0.986, 95% CI: 0.968-1, *P *value: 0.004) (Figure 1).

## Discussion

Toluidine blue, also known by its chemical name tolunium chloride, is a basic metachromatic dye that is known for its property of differentially staining malignant neoplasm but not normal epithelium [14]. It is postulated that the increased amount of DNA and RNA in neoplastic cells and the wider intercellular canals compared to normal epithelial cells are responsible for staining malignant cells [7]. Its clinical application in staining neoplastic cells was first described by Richart in 1963, who used the dye to stain cervical carcinoma *in situ *[15]. Since then it has emerged as a vital stain for detection of cervical dysplasia and carcinoma during colposcopy. More recently toluidine blue has also been described as an adjunct to clinical examination for the detection of malignant and premalignant oral cavity lesions [7,16]. Epstein *et al.*, while screening for recurrence in patients who had previously been treated for upper aero-digestive tract malignancies, found that the use of toluidine blue in an unaided clinical exam increased the sensitivity of detecting a malignant neoplasm from 26.6% to 96.7% [8]. In all fairness its ability to detect malignant oral cavity lesions has been well documented in clinical settings [17,18]. However its use in intraoperative identification of tumor margins has not been well described. Considering its ability to effectively stain malignant cells in contrast to normal epithelium we postulated that it could offer a low cost facility in developing countries, where frozen section is not easily available.

In order to arrive at our conclusion we compared the findings of toluidine blue to frozen section biopsies (standard of care) and final histopathology (gold standard). We found the findings of frozen section biopsies in our sample to be 100% concurrent with the final histopathology. So comparison of toluidine blue with either was equivalent. In our study sample staining with toluidine blue had an intraoperative sensitivity of 100% and a specificity of 97% with a NPV of 100% and a PPV of 27.2%. These figures are in agreement with previous studies done in clinical settings; with slightly higher sensitivity. This could probably be explained by the fact that all the studies quoted above present staining in a clinical scenario whereas our study toluidine blue was used intraoperatively, which allowed a uniform staining protocol to be followed with adequate time for observation

In our study, 11 margins stained positive with toluidine blue, of which three were positive on frozen section biopsy and final histopathology. This low PPV was significant. ROC analysis revealed a *P *value of 0.004. False-positive results have also been shown to be associated with inflammation secondary to increased mucosal trauma during excision [19], indicating that careful handling of the tissue during excision may increase the PPV. However we would highlight the fact that the test had an NPV of 100%, indicating that it did not miss a single positive margin. While reviewing literature we found only one study accessing the use of toluidine blue intraoperatively. In this study, Portugal *et al. *pointed out that staining with toluidine blue identified all positive tumor margins with few false-positives and no false-negatives [20]. Further the study added that staining identified three incidences of second primary lesion that was not previously identified [20]. Missmann *et al. *also reported that toluidine blue staining could not only be used to effectively localize tumor borders but also detect satellite lesions within 1 cm of the primary lesions [21]. We cannot comment on the identification of second primaries, satellite or dysplastic lesions as none were detected in our study.

In resource-limited environments where facilities for frozen section biopsies are not readily available surgeons often rely on either their clinical judgment or on the final postoperative histopathology for assessing microscopic extension of the disease. This leads to a major disadvantage for patients and may account for high recurrence rates of OCSCC in developing countries.

## Conclusion

Coupled with the huge burden of OCSCC in Pakistan [2,22], the risk of recurrence with inadequate excision of the lesion poses a great health and economic challenge to the staggering health system. Given our findings and those reported in literature we feel that toluidine blue can be an effective screening modality for intraoperative margins in resource-limited environments. The number of frozen section biopsies can be limited to the number of positive staining margins, where the facilities of frozen section biopsies are available. The consideration of replacing frozen section biopsies by toluidine blue in resource-limited environments is premature; only with a trial on a larger cohort can such implications be made.

## Competing interests

The authors declare that they have no competing interests.

## Authors' contributions

MJ came up with the idea, wrote the protocol and obtained the ethical committee approval, MC, ZA, MJ did most of the literature review and were mainly involved in the preparing of the manuscript, MK and AS were the primary surgeons and supervised the manuscript writing, SQ and NA were responsible for prepration of dye, obtaining informed consent, application of the dye intra-operatively, observing the results and maintaining the log, GM provided with the necessary statistical input and evaluation of data and results. All authors read and approved the final manuscript.
